# TEMPO-Oxidized Bacterial Cellulose-Stabilized Clove Essential Oil Pickering Emulsions for Sustained-Release Sodium Alginate Active Packaging Films

**DOI:** 10.3390/foods15162924

**Published:** 2026-08-20

**Authors:** Fengge Yu, Jieying Fan, Xiangying Liu, Hongrui Sun, Jialin Zhang, Lining Kang

**Affiliations:** Institute of Agro-Food Technology, Jilin Academy of Agricultural Sciences (Northeast Agricultural Research Center of China), Changchun 130033, China; yufengge2001@163.com (F.Y.);

**Keywords:** TEMPO-oxidized bacterial cellulose, Pickering emulsion, sodium alginate

## Abstract

Developing biodegradable active packaging films that combine mechanical reinforcement, barrier improvement, antibacterial activity, and controlled release of natural antimicrobials remains challenging. Here, TEMPO-oxidized bacterial cellulose (TOBC) was used to stabilize clove essential oil (CEO) Pickering emulsions, which were incorporated into sodium alginate (SA) matrices. The effects of TOBC concentration on emulsion stability and emulsion loading on film-forming solutions, film structure, mechanical performance, barrier properties, antioxidant and antibacterial activities, and CEO release were evaluated. The emulsion stabilized with 0.7 wt% TOBC showed good visual stability after 30 days and was selected for film preparation. Moderate incorporation of the TOBC-stabilized CEO Pickering emulsion improved SA film performance, with SA-E10 showing the best overall balance. SA-E10 reached a tensile strength of 38.56 ± 1.66 MPa and an elongation at break of 10.27%, while the moisture content decreased from 36.48 ± 2.32% to 27.93 ± 0.46%. The films also showed enhanced UV-shielding capacity and lower water vapor permeability. Antioxidant activity increased with emulsion loading, reaching 71.43 ± 2.55% for DPPH and 83.66 ± 1.49% for ABTS. Film-coated paper disks showed visible inhibition zones against *Escherichia coli* and *Staphylococcus aureus*. Compared with direct CEO incorporation, the Pickering emulsion system delayed CEO release, indicating potential for sustained-release active packaging.

## 1. Introduction

Traditional petroleum-based plastic packaging has caused persistent environmental concerns because of its non-biodegradability and long-term accumulation in terrestrial and aquatic ecosystems. In this context, bio-based polymeric materials derived from renewable polysaccharides, proteins, and lipids have attracted increasing attention as sustainable alternatives for food packaging applications owing to their biodegradability, biocompatibility, and film-forming potential [1,2]. Among them, sodium alginate (SA), a natural anionic polysaccharide extracted from brown algae, is considered a promising matrix for biodegradable films because of its good film-forming ability, transparency, biocompatibility, and Generally Recognized as Safe (GRAS) status [3,4]. However, neat SA films usually suffer from limited mechanical strength, poor water vapor barrier performance, and a lack of intrinsic antioxidant and antibacterial activities, which restrict their practical application in active food packaging [5].

Incorporation of natural bioactive compounds into polysaccharide matrices is an effective strategy for endowing packaging films with functional properties. Clove essential oil (CEO), which is rich in eugenol and other phenolic constituents, is attractive for active packaging because of its antioxidant and antibacterial activities [6]. However, its high volatility, hydrophobicity, and limited compatibility with hydrophilic polymer matrices may lead to uneven dispersion and rapid activity loss during film preparation and storage [7]. These limitations make it necessary to develop a delivery strategy that improves CEO dispersion, reduces volatilization, and regulates its release from the film matrix.

Pickering emulsions, which are stabilized by solid particles rather than conventional molecular surfactants, provide a promising platform for incorporating hydrophobic essential oils into hydrophilic biopolymer matrices [8]. The adsorption of particles at the oil–water interface can form a physical barrier around oil droplets, thereby reducing droplet coalescence and improving emulsion stability [9]. When incorporated into film-forming systems, Pickering emulsion droplets may also act as dispersed functional domains, increasing the tortuosity of diffusion pathways and contributing to the controlled release of essential oils [10]. Therefore, Pickering emulsion-based incorporation is particularly suitable for constructing active packaging films with improved dispersion stability, barrier properties, and sustained bioactivity.

The performance of Pickering emulsions depends strongly on the properties of the particulate stabilizer. Bacterial cellulose (BC), a nanofibrous cellulose produced by microbial fermentation, possesses high purity, a large specific surface area, and excellent mechanical properties, making it a promising bio-based particle for emulsion stabilization [11]. However, pristine BC is highly hydrophilic and tends to aggregate in aqueous media due to extensive hydrogen bonding, which limits its interfacial adsorption at the oil–water interface [12]. TEMPO-mediated oxidation can selectively introduce carboxyl groups onto the surface of BC nanofibers, producing TEMPO-oxidized bacterial cellulose (TOBC) with improved colloidal dispersibility, surface charge, and interfacial activity [13,14,15]. These features are expected to facilitate the adsorption of TOBC at the CEO–water interface and promote the formation of a stabilizing particle layer and a cellulose-based network in the continuous phase [16].

Sodium alginate is an established film-forming polysaccharide, and its selection alone is not the novelty of this study [3]. The distinguishing feature of the present design is the connected dual role of TOBC. First, TOBC acts as the particulate stabilizer at the CEO–water interface, improving the dispersion and retention of the hydrophobic oil. Second, after the emulsion is incorporated and the film is cast, the TOBC-coated droplets and dispersed nanofibers contribute to the organization of the SA matrix and can modify its mechanical, barrier, and release behavior. Accordingly, this work focuses on how TCPE loading governs the balance among film reinforcement, water vapor resistance, bioactivity, and sustained CEO release rather than presenting SA itself as a new film material.

Therefore, the objective of this study was to develop multifunctional SA-based active packaging films by incorporating TOBC-stabilized clove essential oil Pickering emulsions (TCPEs). The stability and microstructure of the TCPEs were first evaluated, and the effects of TCPE incorporation on the rheological behavior of film-forming solutions, film morphology, mechanical properties evaluated according to ISO 527-3:2018 [17], water vapor barrier performance, UV-shielding capacity, antioxidant and antibacterial activities, and CEO release behavior were systematically investigated. This study aims to clarify how TOBC-stabilized Pickering emulsions regulate both film structure and functional performance, providing a feasible strategy for designing biodegradable active packaging films with sustained-release capability.

## 2. Materials and Methods

### 2.1. Materials

Bacterial cellulose (BC) was produced by *Komagataeibacter saccharivorans* LYJG-1 according to the method reported by Yu et al. [11]. 2,2,6,6-Tetramethylpiperidine-1-oxyl (TEMPO), sodium bromide, sodium hypochlorite solution, ethanol, hydrochloric acid, sodium hydroxide, and sodium alginate were purchased from Sinopharm Chemical Reagent Co., Ltd. (Shanghai, China). Clove essential oil (CEO) was obtained from Sigma-Aldrich (St. Louis, MO, USA). Glycerol, Nile Red, and Calcofluor White were purchased from Aladdin Reagent Co., Ltd. (Shanghai, China). Unless otherwise specified, all reagents were of analytical grade and used without further purification. *Staphylococcus aureus* ATCC 25923 and *Escherichia coli* ATCC 25922 were used as Gram-positive and Gram-negative model bacteria, respectively, for antibacterial assays.

### 2.2. Preparation of TEMPO-Oxidized Bacterial Cellulose (TOBC)

TOBC was prepared following the methodology reported by Dong et al. [18]. Briefly, BC was dispersed in deionized water at a ratio of 100 mL per gram of dry BC. TEMPO and sodium bromide were added at 0.1 mmol/g and 1 mmol/g (based on dry BC weight), respectively, and the mixture was stirred thoroughly to obtain a uniform suspension. The suspension was then homogenized using an ULTRA-TURRAX T25 basic homogenizer (IKA, Staufen, Germany) at 10,000 rpm for 4 min to obtain a homogeneous BC suspension. An aqueous sodium hypochlorite solution (2.0 mol/L NaClO; 1.0 mL per gram of dry BC, corresponding to 2.0 mmol NaClO/g dry BC) was added dropwise at room temperature to initiate oxidation. The reaction was continued for 2 h, while the pH was maintained at approximately 10 by gradual addition of 0.1 mol/L NaOH. At the end of the reaction, 2.0 mL of anhydrous ethanol per gram of dry BC was added to terminate the oxidation. The oxidized product was collected by centrifugation at 10,000 rpm for 10 min, washed repeatedly with deionized water, and dialyzed against deionized water for 24 h using a membrane with a molecular weight cutoff (MWCO) of 12–14 kDa to remove residual ions and low-molecular-weight reaction products. The resulting TOBC was stored at 4 °C until further use. Conductivity titration indicated that the carboxyl group content of TOBC was 0.9 mmol/g.

### 2.3. Preparation of the TEMPO-Oxidized Bacterial Cellulose-Stabilized Pickering Emulsion

TOBC-stabilized clove essential oil Pickering emulsions (TCPE) were prepared by homogenizing the aqueous TOBC suspension with clove essential oil (CEO) [19]. Briefly, TOBC suspensions with different concentrations of 0.1, 0.3, 0.5, 0.7, and 0.9 wt% were prepared in deionized water. Each TOBC suspension was then mixed with CEO at an aqueous phase/oil phase mass ratio of 8:2, corresponding to an oil phase fraction of 20 wt%. The mixtures were homogenized using a high-speed homogenizer (FA25-20DG, FLUKO, Shanghai, China) at 12,000 rpm for 5 min at room temperature to obtain TOBC-stabilized CEO Pickering emulsions. The prepared emulsions were sealed and stored at 4 °C before subsequent characterization and film preparation.

### 2.4. Characterization of TOBC and TCPE

#### 2.4.1. Atomic Force Microscopy (AFM)

The morphological features of TOBC were examined using atomic force microscopy. The TOBC suspension was diluted to 0.01 wt% with ultrapure water and deposited onto freshly cleaved mica substrates. After air-drying at ambient temperature, the samples were mounted on the AFM stage (Dimension Icon, Bruker, Santa Barbara, CA, USA) and imaged in tapping mode. Fiber dimensions and height profiles were analyzed using Nanoscope Analysis 1.8 software.

#### 2.4.2. Transmission Electron Microscopy (TEM)

The microstructure of TOBC nanofibers was further observed using transmission electron microscopy (JEM-1200EX, Jeol Ltd., Tokyo, Japan). TOBC suspensions (0.008% *w*/*w*) were ultrasonicated for 30 min to ensure adequate dispersion, dropped onto copper grids, and stained with 1.5% (*w*/*w*) phosphotungstic acid for 15 min in the dark. Fiber diameters were measured from 100 randomly selected nanofibers, and the results were used to construct a diameter distribution histogram.

#### 2.4.3. Fourier-Transform Infrared (FTIR)

FTIR analysis was performed using a Nicolet Summit X spectrometer (Thermo Fisher Scientific, Waltham, MA, USA) to evaluate the chemical structure of TOBC. Samples were mixed thoroughly with potassium bromide (KBr) and pressed into pellets. Spectra were collected in transmission mode over the range of 500–4000 cm^−1^ with a resolution of 4 cm^−1^, averaging 32 scans.

#### 2.4.4. X-Ray Photoelectron Spectroscopy (XPS)

XPS measurements were conducted using an ESCALAB 250 spectrometer (Thermo Fisher Scientific, East Grinstead, UK) equipped with an Al Kα X-ray source. Dried TOBC samples were analyzed directly without any pretreatment. High-resolution spectra were obtained for the C 1s and O 1s regions to characterize surface chemical compositions and oxidation states.

#### 2.4.5. Confocal Laser Scanning Microscopy (CLSM)

The microstructure of the TOBC-stabilized Pickering emulsion was visualized by confocal laser scanning microscopy (CLSM, FV3000, Olympus Corporation, Tokyo, Japan) following a modified protocol from Hou et al. [19]. A mixed dye solution containing 0.1 wt% Nile Red and 1 wt% Calcofluor White was added to the emulsion at a ratio of 20 μL per 1 mL of emulsion. Nile Red was used to stain the oil phase, whereas Calcofluor White was used to label the cellulose nanofibers. The excitation wavelengths were 535 nm for Nile Red and 405 nm for Calcofluor White.

#### 2.4.6. Apparent Droplet Size

The apparent droplet size distribution of TCPE was measured using a Zetasizer Nano ZSE instrument (Malvern Panalytical, Malvern, UK). Before measurement, the emulsions were gently diluted with deionized water at a ratio of 1:100 to reduce multiple scattering effects. The diluted samples were mixed carefully by gentle inversion, and no sonication was applied before measurement to avoid disrupting the original emulsion droplets. The results were expressed as apparent hydrodynamic droplet diameter and used for comparative analysis among different TCPE formulations.

#### 2.4.7. Macroscopic Stability of Emulsions

The long-term macroscopic stability of TCPE was evaluated by storing the emulsion in glass cylinders with graduation marks at room temperature for 30 days [15]. Throughout the storage period, the emulsions were visually monitored for signs of creaming, phase separation, or sedimentation. Stability was assessed based on the uniformity of the emulsion and the absence of visible structural changes or separated layers.

### 2.5. Preparation of Composite Film

Sodium alginate powder was dispersed in deionized water to obtain a 1.5% (*w*/*w*) solution. The mixture was heated in a 25 °C water bath and subjected to magnetic stirring until complete dissolution, yielding a clear and uniform film-forming solution. Glycerol, added at 20% of the sodium alginate mass, was introduced as a plasticizer and stirred thoroughly to ensure homogeneous incorporation, resulting in a stable sodium alginate matrix.

Based on the emulsion stability results, the TCPE stabilized with 0.7 wt% TOBC was selected for film preparation. The selected TCPE was incorporated into the sodium alginate (SA) film-forming solution at concentrations of 0%, 5%, 10%, and 15% (*w*/*w*). The mixtures were stirred at 25 °C for 30 min and vacuum-degassed for 5 min. For each formulation, 15 mL of film-forming solution was poured into a horizontally leveled PTFE mold with internal dimensions of 10 × 10 cm. The films were dried under static conditions at 25 °C and 50% relative humidity for 24 h, carefully peeled from the molds, and equilibrated at 25 °C and 50% relative humidity for 48 h before storage in a desiccator. According to the TCPE content, the films were designated as SA (0 wt%), SA-E5 (5 wt%), SA-E10 (10 wt%), and SA-E15 (15 wt%).

### 2.6. Rheological Measurements of Film-Forming Fluids

A 1 mL aliquot of each film-forming solution was transferred onto the plate of a rotational rheometer (MCR 302, Anton Paar GmbH, Graz, Austria) maintained at 25 °C. Measurements were conducted using a 40 mm parallel-plate geometry with a fixed 1 mm gap. Steady-state shear tests were carried out over a shear-rate range of 0.1–100 s^−1^ to investigate the flow characteristics of the solutions. Additionally, dynamic frequency sweep tests were performed at a constant strain of 1%, with angular frequencies ranging from 0.1 to 100 Hz, to determine the viscoelastic behavior of the film-forming systems.

### 2.7. Characterization of Film Properties

#### 2.7.1. Scanning Electron Microscopy (SEM)

The surface morphology of the films was examined using a Mira 4 SEM (Tescan, Brno, Czech Republic). Samples were sectioned, mounted onto aluminum stubs using conductive aluminum adhesive tape, and sputter-coated with a thin layer of gold to enhance electrical conductivity.

#### 2.7.2. FTIR Spectroscopy

FTIR spectra were collected using a Nicolet Summit X spectrometer (Thermo Fisher Scientific, Waltham, MA, USA) equipped with an ATR accessory. Spectral data were recorded from 400 to 4000 cm^−1^ at a resolution of 4 cm^−1^, and each spectrum represented the average of 32 scans.

#### 2.7.3. X-Ray Diffraction (XRD) Analyzer

XRD patterns of the films were obtained using an X-ray diffractometer (SmartLab, Rigaku Corporation, Tokyo, Japan). Data were recorded over a 2θ range of 10–60° with a scanning rate of 5°/min.

#### 2.7.4. Thermogravimetric Analysis (TGA)

Thermal stability was assessed using a thermogravimetric analyzer (STA449F5, Netzsch- Gerätebau GmbH, Selb, Germany). Approximately 4 mg of each film sample was placed in a quartz crucible and heated from 30 °C to 600 °C at a constant rate of 10 °C/min under a continuous nitrogen (N_2_) flow of 20 mL/min.

#### 2.7.5. Mechanical Properties

Before testing, the films were equilibrated at 25 °C and 50% relative humidity for 48 h. Mechanical properties were determined according to ISO 527-3:2018 [17] using a reduced-size dumbbell specimen and a universal tensile tester (AGS-X, Shimadzu Corporation, Kyoto, Japan). Dumbbell-shaped specimens with a total length of 75 mm, a narrow-section width of 5 mm, and a gauge length of 25 mm were stretched at a crosshead speed of 20 mm/min. Tensile strength and elongation at break were calculated from three replicates and reported as mean values.

#### 2.7.6. Moisture Content (MC), Water Solubility (WS) and Water Vapor Permeability (WVP)

The mean thicknesses of the SA, SA-E5, SA-E10, and SA-E15 films were 0.061 ± 0.003, 0.067 ± 0.002, 0.074 ± 0.003, and 0.082 ± 0.002 mm, respectively. Film samples (2 cm × 2 cm) were weighed (M_1_) and dried at 105 °C until a constant mass (M_2_) was obtained. The dried samples were immersed in 10 mL of deionized water at 25 °C for 24 h. The undissolved residues were collected, dried again at 105 °C, and weighed as M_3_. Moisture content and water solubility were calculated using the following equations [14]:

(1) $$\mathrm{MC}\;\left(\%\right)=\left(M_{1}-M_{2}\right)/M_{1}\times 100\%\;\mathrm{WS}\;\left(\%\right)=\left(M_{2}-M_{3}\right)/M_{2}\times 100\%$$

WVP values were determined using the sealed cup method. Briefly, 3.0 g of anhydrous calcium chloride was placed inside each test cup to maintain a low relative humidity on the inner side of the film. The film sample was tightly fixed over the cup opening and sealed to prevent edge leakage. The cups were then placed in a chamber maintained at 25 °C and 75% relative humidity, thereby establishing a water vapor pressure gradient across the film. Mass changes were recorded every 24 h for four consecutive days. WVP was calculated using the following equation:

(2) $$WVP=\frac{\Delta m\times L}{A\times t\times \Delta P}$$

where Δm is the mass change (g), L is the measured thickness of each film specimen (mm), A is the testing area (m^2^), t is the measurement time (h), and ΔP is the vapor pressure gradient across the film.

#### 2.7.7. Ultraviolet Transmittance Measurement

Rectangular specimens were used to assess optical properties. The UV transmittance of the films was measured from 200 to 400 nm using a UV–visible spectrophotometer (UV-1780, Shimadzu Corporation, Kyoto, Japan), with air serving as the reference.

#### 2.7.8. Sustained-Release Effect of the Film

The release profile of clove essential oil (CEO) from the composite films was evaluated using a UV spectrophotometer at 282 nm. Film samples (15 mm × 15 mm; exposed area ≈ 2.25 cm^2^) from SA-E10 and a control film containing an equivalent amount of directly added CEO were accurately weighed and immersed in 40 mL of 50% (*v*/*v*) ethanol inside 50 mL centrifuge tubes. Samples were incubated in a constant-temperature shaker (25 ± 0.5 °C, 100 rpm). At predetermined intervals, 100 μL aliquots were withdrawn and replenished with fresh medium. CEO concentrations were quantified using a previously established calibration curve. The theoretical maximum CEO loading remained well below its solubility limit (0.2 g/mL) in 50% ethanol, ensuring non-saturation conditions throughout the release test.

#### 2.7.9. Antibacterial and Antioxidant Activities of the Films

Antibacterial activity was qualitatively assessed using the agar disk diffusion method against *Staphylococcus aureus* (ATCC 25923) and *Escherichia coli* (ATCC 25922), following the procedure reported by Yang et al. [14]. Sterile filter paper disks with a diameter of 6 mm were immersed in the film-forming solutions for 5 min, removed, and then dried under aseptic conditions to obtain dried film-coated paper disks. Paper disks coated with the pure SA solution and sterile water-treated paper disks were used as controls. The dried coated disks were placed onto nutrient agar plates inoculated with 0.1 mL of bacterial suspension (10^7^ CFU/mL). After incubation at 37 °C for 24 h, the inhibition zones around the disks were recorded.

Antioxidant capacity was evaluated via DPPH and ABTS radical scavenging assays according to the method of Jia et al. [20]. For the DPPH test, 20.0 mg of film was mixed with 2.0 mL anhydrous ethanol and 2.0 mL of DPPH solution (2.0 × 10^−4^ mol/L) and incubated in the dark for 30 min before measuring absorbance at 517 nm. For the ABTS assay, 10.0 mg of film was mixed with 8.0 mL ABTS working solution for 3 min, and absorbance at 734 nm was recorded. Radical scavenging activity was calculated using:

(3) $$\mathrm{DPPH}/\mathrm{ABTS}\;\mathrm{radical}\;\mathrm{scavenging}\;(\%)=\frac{A-A_{i}}{A}\times 100\%$$

where A refers to the absorbance of the blank solutions at 517 or 734 nm, and A_i_ is the absorbance of the film samples.

### 2.8. Statistical Analysis

All experiments were performed in triplicate, and results are expressed as mean ± standard deviation (SD). Statistical analyses were conducted using SPSS 18.0, with significance defined as *p* < 0.05. Data visualizations were produced using Origin 8.5.

## 3. Results and Discussion

### 3.1. Characterization of TEMPO-Oxidized Bacterial Cellulose (TOBC)

#### 3.1.1. Morphological Observation

**Figure 1:**
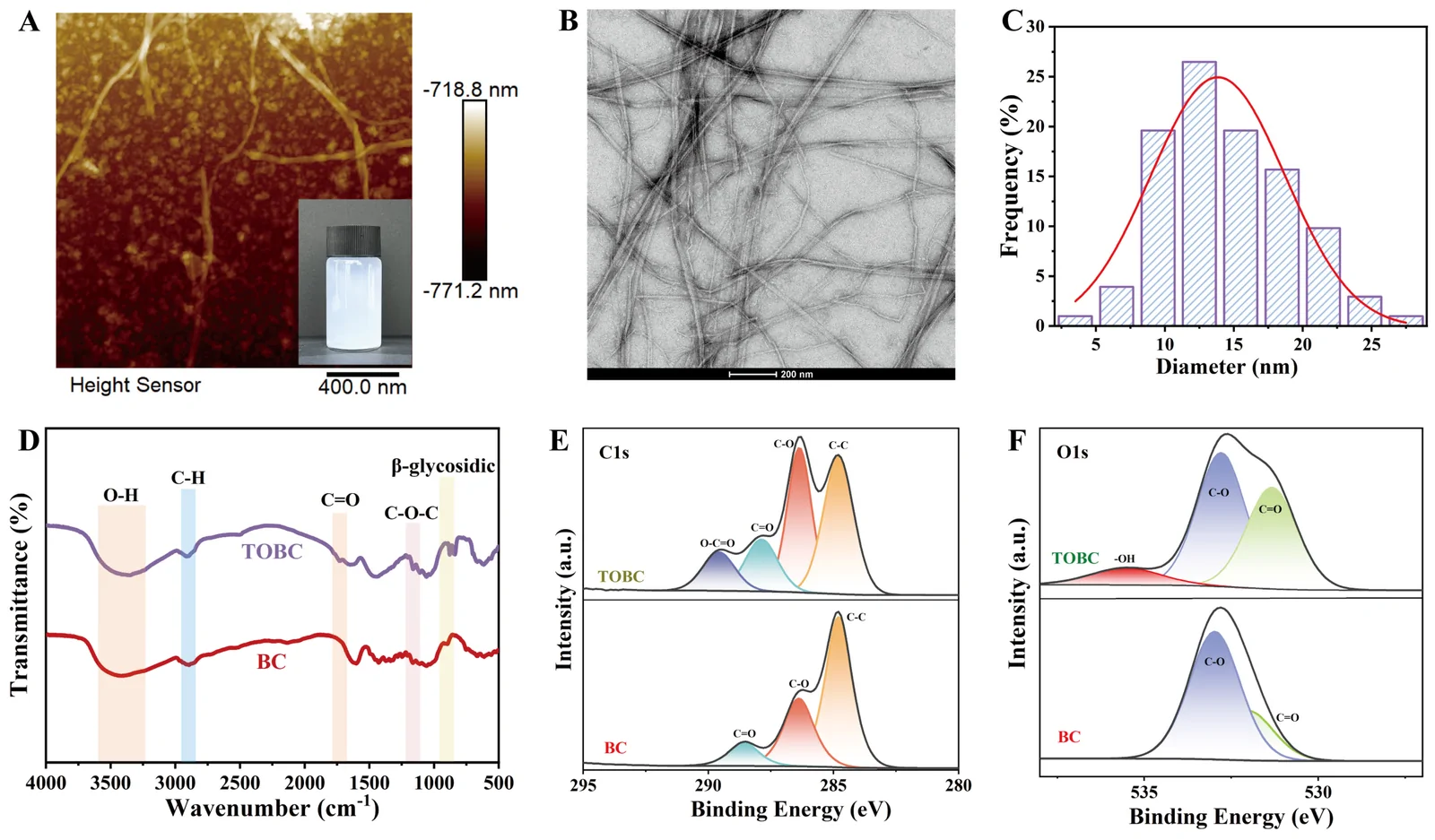
(**A**) AFM image of TEMPO-oxidized bacterial cellulose (TOBC); the inset shows the corresponding aqueous TOBC suspension used for AFM analysis. (**B**) TEM image illustrating the morphology of TOBC nanofibers. (**C**) Diameter distribution of TOBC nanofibers, determined by randomly measuring 100 individual fibrils from the TEM image. (**D**) FTIR spectra of bacterial cellulose (BC) and TOBC. (**E**) High-resolution XPS spectra of the C1s region for TOBC and BC. (**F**) High-resolution XPS spectra of the O1s region for TOBC and BC.

Figure S1 presents a scanning electron microscopy (SEM) image of the pristine bacterial cellulose (BC) membrane, revealing densely interwoven fibers with localized agglomeration, indicative of a compact structure and limited dispersion. Following TEMPO-mediated selective oxidation, the resulting TOBC displays distinctly altered morphological characteristics. Atomic force microscopy (AFM) images (Figure 1A) demonstrate that TOBC is composed of uniformly dispersed nanofibers, forming a loosely interconnected yet continuous network with no visible aggregation. The surface exhibits characteristic fibrous textures, indicating effective fibrillation and improved dispersion after oxidation. This pronounced improvement in fiber dispersion is primarily attributed to the carboxyl groups introduced by TEMPO oxidation, which confer negative charges on the fiber surfaces, generating electrostatic repulsion that mitigates nanofiber reaggregation [21]. The AFM inset shows that the TOBC suspension was transparent and macroscopically homogeneous.

Transmission electron microscopy (TEM) images (Figure 1B) provide additional insight into the nanostructure of TOBC. Post-oxidation, fibers are well-separated, exhibiting elongated and continuous morphologies with clearly defined edges and minimal particle adhesion. Some fibers display slight curvature, indicating inherent flexibility. Quantitative analysis was conducted on 100 randomly selected fiber segments, and the resulting diameter distribution histogram is shown in Figure 1C. The analysis reveals that TOBC nanofiber diameters predominantly fall within the 10–20 nm range, with an average of 14.64 ± 4.75 nm, indicating relatively uniform fiber dimensions. These observations are consistent with both AFM and TEM data, demonstrating that TEMPO oxidation not only facilitates single-fiber separation of BC microfibers but also significantly enhances uniformity in fiber dispersion.

#### 3.1.2. TOBC Chemical Structure Analysis

FTIR spectra of BC and TOBC (Figure 1D) retained the principal cellulose bands, including O–H stretching at 3400–3200 cm^−1^, C–H stretching near 2900 cm^−1^, and the β-glycosidic vibration at 890 cm^−1^. After TEMPO oxidation, a carbonyl band emerged at 1730 cm^−1^, which is attributable to carboxyl groups introduced at the C6 position. This assignment is consistent with a recent FTIR characterization of TEMPO-oxidized bacterial cellulose [18]. The change in the width and intensity of the O–H region is more appropriately interpreted as reorganization of the hydrogen-bonding environment than as direct quantitative evidence of hydroxyl consumption. Meanwhile, the persistence of the C–H and β-glycosidic bands indicates that the main cellulose framework was retained.

XPS provided complementary surface-sensitive evidence of oxidation (Figure 1E,F). In the C1s spectrum, the increased O–C=O and C=O contributions after oxidation are consistent with the formation of surface carboxyl groups [22]. The O1s spectrum likewise showed a greater contribution from carbonyl-containing species and a high-binding-energy component associated with carboxylic acid hydroxyls. Because XPS primarily probes the near-surface region, these changes support surface carboxylation rather than deep oxidation of the fibril interior. The relatively stable C–O contribution, together with the retained cellulose bands in FTIR and the measured carboxyl content of 0.9 mmol/g, indicates selective oxidation while preserving the principal cellulose backbone.

### 3.2. Characterization of Pickering Emulsion

#### 3.2.1. Observation by Confocal Laser Scanning Microscopy (CLSM)

**Figure 2:**
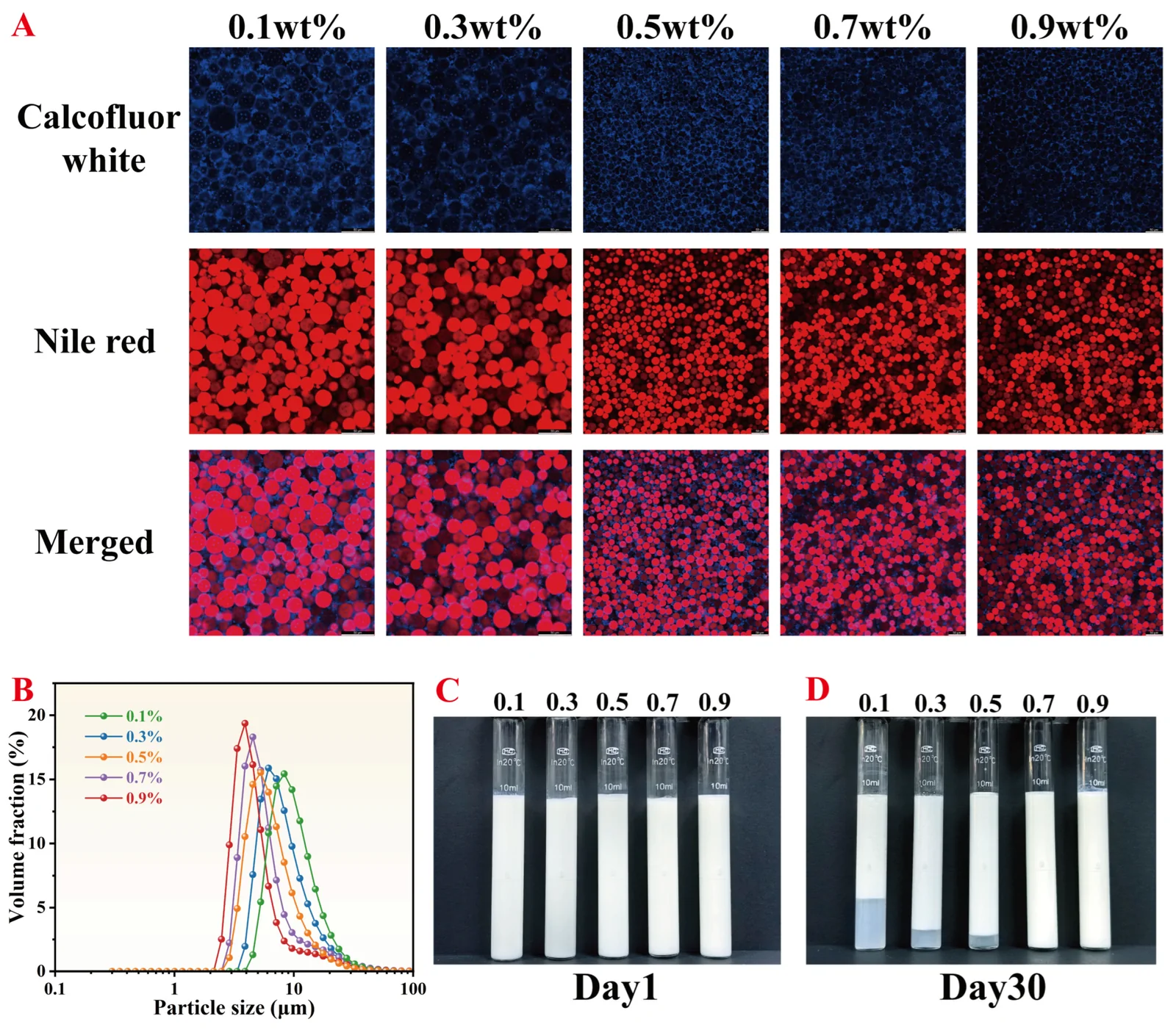
(**A**) CLSM images of Pickering emulsions stabilized by TOBC at different concentrations (0.1–0.9 wt%), using Calcofluor White (blue) and Nile Red (red) staining; merged images highlight interactions between TOBC and the oil phase. (**B**) Droplet size distribution of Pickering emulsions as a function of TOBC concentration, showing volume fraction (%) versus particle size (µm). (**C**) Macroscopic appearance of emulsions on Day 1. (**D**) Macroscopic appearance of emulsions on Day 30. Scale bars in the CLSM images: 50 μm.

The distribution of TOBC within the emulsion was directly visualized using CLSM (Figure 2A). Results reveal that TOBC concentration exerts a profound influence on the emulsion microstructure. At low concentrations (0.1 wt%), Nile Red-labeled oil droplets exhibit larger diameters and poor dispersion, while the blue fluorescence from Calcofluor White primarily appears as discrete spots, indicating insufficient interfacial adsorption. At intermediate concentrations (0.3–0.5 wt%), oil droplet diameters markedly decrease, showing narrower distributions, with more continuous blue ring structures forming along droplet surfaces. At higher concentrations (0.7–0.9 wt%), nearly all oil droplets are encircled by complete blue rings, and the blue fluorescence in the continuous phase interconnects to form a three-dimensional network.

These observations highlight the dual stabilization mechanism of TOBC. Firstly, the “blue ring” surrounding the oil droplets demonstrates that TOBC, acting as Pickering particles, forms a robust interfacial barrier, effectively preventing droplet coalescence. Comparable interfacial ring structures have been reported for cellulose nanocrystal-stabilized emulsions, supporting the interpretation of “particle adsorption–interface blocking” [23]. Secondly, the fully interconnected cellulose network observed at higher concentrations indicates that excess TOBC self-assembles in the aqueous phase, generating steric hindrance and viscoelastic constraints that restrict droplet motion and aggregation. Previous studies have confirmed the positive correlation between macro-/micro-rheology and emulsion stability in TOBC systems, demonstrating that network reinforcement substantially enhances resistance to flocculation and phase separation [24]. Moreover, the plateau in droplet size at high concentrations indicates that interfacial adsorption reaches saturation, with surplus TOBC primarily reinforcing the bulk three-dimensional network [25].

#### 3.2.2. Particle Size Distribution

Particle size analysis (Figure 2B) confirms that TOBC concentration significantly modulates droplet size distribution in clove essential oil Pickering emulsions. Increasing TOBC content from 0.1 wt% to 0.9 wt% progressively reduces the average droplet size, which stabilizes at higher concentrations (0.7–0.9 wt%). At 0.1 wt%, droplets are largest with a broad, non-uniform distribution, reflecting inadequate interfacial coverage. Under homogenization, transient droplet breakup may occur; however, short-range attractions between under-covered droplets promote mild flocculation, resulting in maximum particle sizes and distribution widths [26]. This behavior is consistent with other cellulose nanoparticle systems during the under-coverage stage [27].

At intermediate concentrations (0.3–0.5 wt%), increased interfacial adsorption forms a continuous particle layer, lowering interfacial energy and generating a geometric barrier that synergizes with steric and electrostatic repulsion. Consequently, the main peak shifts toward smaller sizes, and the distribution narrows, yielding finer and more uniform droplets [23]. Further increasing TOBC to 0.7–0.9 wt% produces minimal additional shifts in the main peak, forming a plateau, indicative of interfacial saturation: additional particles primarily enter the continuous phase to form a bulk three-dimensional cellulose network. At this stage, droplet size is dictated by homogenization energy and the viscoelastic properties of the continuous phase rather than particle availability [28], corroborating CLSM observations.

#### 3.2.3. Macroscopic Stability of Emulsions

Macroscopic stability reflects an emulsion’s ability to resist phase separation and maintain homogeneity. As shown in Figure 2C,D, TOBC concentration critically influences emulsion stratification. After 1 day, no flocculation is observed in any sample. After 30 days, emulsions with low TOBC concentrations (0.1, 0.3, 0.5 wt%) exhibit significant phase separation, whereas high-concentration samples (0.7–0.9 wt%) remain uniform, demonstrating that elevated TOBC content enhances long-term stability.

This macroscopic behavior can be rationalized from a microstructural perspective. Emulsion stability is closely linked to droplet size and network architecture: at low TOBC concentrations, incomplete interfacial coverage results in coarse, polydisperse droplets prone to rapid coalescence and flotation. In contrast, at higher concentrations, the formation of a complete interfacial “particle armor” and an interconnected three-dimensional cellulose network imposes steric hindrance, effectively impeding droplet migration and aggregation, thus conferring superior stability [29].

These findings are consistent with prior studies. Wang et al. and He et al. reported that increasing the concentration of cellulose-based emulsifiers enhances emulsion stability by promoting interfacial adsorption saturation and bulk network formation [30,31]. In the present study, emulsions stabilized with 0.7–0.9 wt% TOBC showed good resistance to gravitational separation during 30 days of storage, indicating their potential applicability in active packaging systems. Considering the comparable long-term stability of the emulsions stabilized with 0.7 and 0.9 wt% TOBC, the 0.7 wt% TOBC-stabilized TCPE was selected for subsequent film preparation to achieve effective emulsion stabilization while minimizing stabilizer usage.

### 3.3. Rheological Properties of Film-Forming Liquids

#### 3.3.1. Steady-State Shear

**Figure 3:**
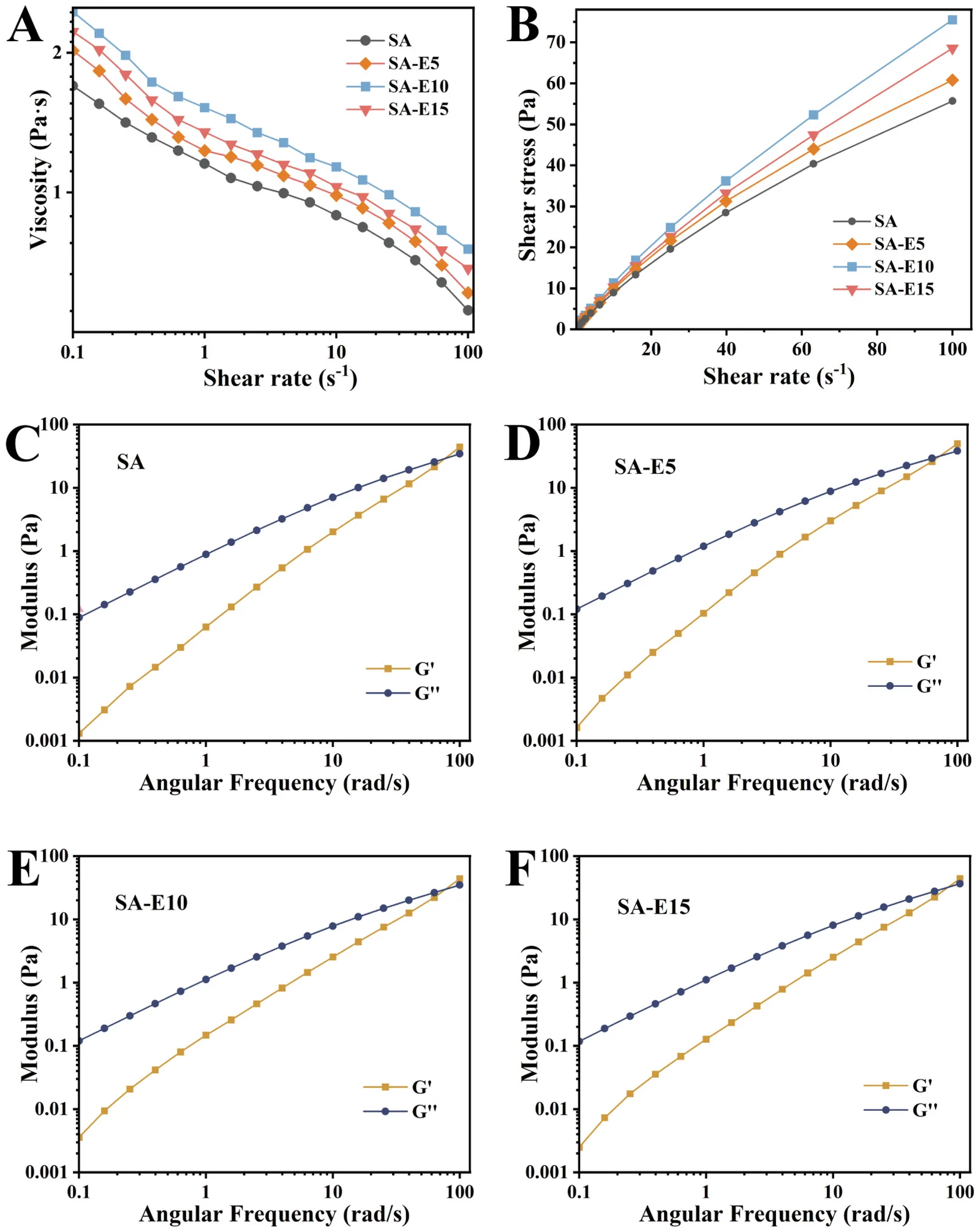
(**A**) Shear-rate-dependent viscosity of film-forming solutions containing varying TCPE concentrations (0%, 5%, 10%, 15%). (**B**) Shear stress profiles of the same solutions as a function of shear rate. (**C**) Frequency sweep of the pure sodium alginate (SA) solution, showing storage modulus (G′) and loss modulus (G″). (**D**) Frequency sweep of the SA solution containing 5% TCPE. (**E**) Frequency sweep of the SA solution containing 10% TCPE. (**F**) Frequency sweep of the SA solution containing 15% TCPE.

The steady-state shear viscosity of the film-forming solutions is presented in Figure 3A. All samples exhibit a pronounced decrease in viscosity with increasing shear rate, characteristic of pseudoplastic fluids, i.e., shear-thinning behavior. This phenomenon is attributed to the alignment of molecular chains under applied shear and the concomitant reduction in intermolecular friction [32]. The incorporation of TCPE (TEMPO-oxidized bacterial cellulose-stabilized Pickering emulsion) notably increases the initial viscosity of the solutions. This enhancement likely arises from physical entanglements between TOBC within the TCPE continuous phase and sodium alginate (SA) chains [33].

However, a reduction in viscosity is observed when the TCPE content reaches 15%. Liu et al. suggested that excessive emulsion addition can promote the formation of lipid–polysaccharide complexes via aggregation and interactions between the polysaccharide matrix and emulsion droplets, thereby reducing viscosity [34]. Wang et al. further proposed that the observed rheological behavior arises from two competing mechanisms: at low TCPE concentrations, the thickening effect of TOBC dominates, slightly increasing viscosity; whereas at higher concentrations, the disruptive influence of emulsion droplets on the polymer network prevails, leading to viscosity reduction. Correspondingly, shear stress measurements (Figure 3B) exhibit trends consistent with the observed viscosity evolution following TCPE incorporation.

#### 3.3.2. Frequency Scanning

The dynamic rheological properties of the film-forming solutions critically affect both processing stability and the mechanical performance of the resulting films. Frequency sweep tests were conducted to systematically evaluate the impact of varying TCPE content on the viscoelastic behavior of SA film-forming solutions (Figure 3C–F). All solutions display typical frequency-dependent behavior: both the storage modulus (G′) and loss modulus (G″) increase with angular frequency. In the low-frequency regime, G″ exceeds G′, indicating liquid-like (viscous) behavior, which reflects sufficient molecular relaxation and chain rearrangement over extended timescales. As frequency increases, G′ surpasses G″, demonstrating solid-like (elastic) behavior, attributable to limited relaxation time and the dominance of transient molecular entanglements and interactions [34].

Notably, increasing the TCPE content from 0% to 10% shifted the crossover point of G′ and G″ toward lower frequencies, indicating longer relaxation times and enhanced network stability [35]. TOBC nanofibers and emulsion droplets may act as dispersed physical junctions within the SA matrix. Hydrogen bonding between TOBC and SA chains, together with electrostatic repulsion among their deprotonated carboxylate groups, can improve nanofiber dispersion and suppress local aggregation [14]. Combined with chain entanglement and droplet-induced confinement, these effects may support a more stable transient network and promote the transition from predominantly fluid-like behavior to weak gel-like behavior.

However, at 15% TCPE, the crossover point shifts back to higher frequencies, signifying a disruption of network integrity due to excessive emulsion addition. This observation aligns with the “excess emulsion-induced network weakening” effect reported in konjac glucomannan/Pickering emulsion composite systems [36].

### 3.4. Characterization of Films

#### 3.4.1. Scanning Electron Microscopy (SEM) Analysis

**Figure 4:**
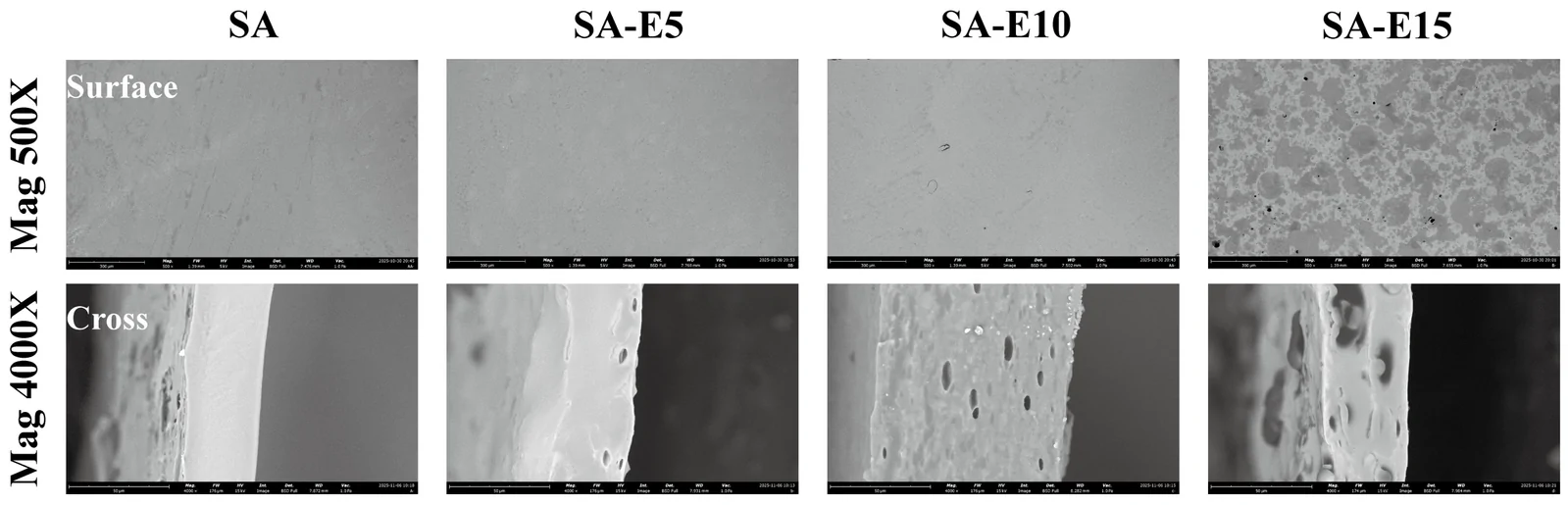
SEM images of SA films incorporating different concentrations of TCPE. SA: Surface and cross-sectional views of the pure SA film; SA-E5: Surface and cross-sectional views of SA film with 5% TCPE; SA-E10: Surface and cross-sectional views of SA film with 10% TCPE; SA-E15: Surface and cross-sectional views of SA film with 15% TCPE. Surface images were captured at 500× magnification, while cross-sectional images were taken at 4000× magnification.

SEM images of the film surfaces and cross-sections are shown in Figure 4. The pure SA film exhibited a relatively smooth surface and a dense, continuous cross-sectional structure without obvious pores, indicating good structural integrity. After incorporation of TCPE, gradual changes in film morphology were observed. The SA-E5 film still maintained a relatively uniform and continuous structure, suggesting good compatibility between the low-concentration Pickering emulsion and the SA matrix. However, when the TCPE content increased to 10%, noticeable voids and irregular pore structures appeared in the cross-section, accompanied by localized structural heterogeneity. These features were likely associated with partial aggregation of emulsion droplets within the matrix during film formation. In addition, several circular protrusions were observed on the film surface, which may be attributed to migration of TCPE droplets during water evaporation and drying [37]. At 15% TCPE loading, the structural heterogeneity became more pronounced, resulting in larger pore structures and a looser internal architecture. The film surface also became rougher, indicating reduced structural uniformity at excessive emulsion concentrations. Similar phenomena have been reported by Zhang et al. [38], who found that high concentrations of Pickering emulsions in sodium alginate films promoted droplet collision and coalescence, thereby adversely affecting film morphology.

#### 3.4.2. Fourier Transform Infrared (FTIR) Analysis of Films

**Figure 5:**
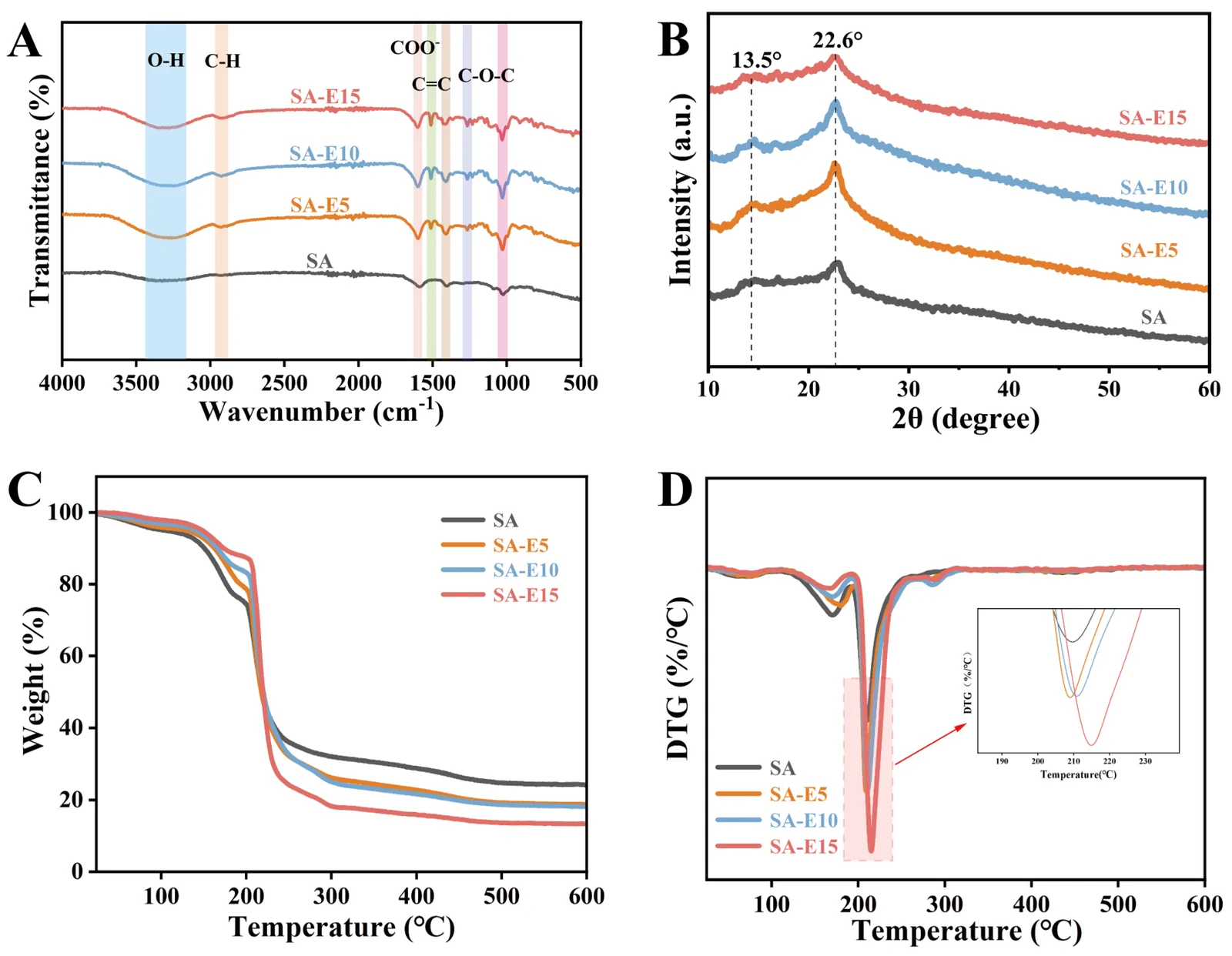
Characterization of SA films containing different TCPE concentrations. (**A**) FTIR spectra of films with 0%, 5%, 10%, and 15% TCPE. (**B**) XRD patterns of films with 0%, 5%, 10%, and 15% TCPE. (**C**) TGA curves of films with varying TCPE content. (**D**) DTG curves corresponding to (**C**), with the inset highlighting the temperature range around 200 °C.

FTIR spectra (Figure 5A) reveal molecular interactions and structural changes in the film matrix. Pure sodium alginate exhibits characteristic absorption peaks at 3320 cm^−1^ (O–H stretching), 1615 cm^−1^ and 1410 cm^−1^ (COO− symmetric and asymmetric stretching), and 1023 cm^−1^ (glycosidic C–O–C vibration) [39]. In composite films, new peaks at 1510 cm^−1^ and 1268 cm^−1^ correspond to aromatic C=C and aromatic ether C–O–C stretching vibrations of eugenol, providing spectroscopic evidence for eugenol-related functional groups and supporting the presence of CEO-derived components in the composite films [40]. All composite films exhibited red-shifted O–H bands with broadened peak shapes, indicative of hydrogen bonding between emulsion components and sodium alginate chains [41], consistent with Xu et al. [42], who observed similar shifts in Pickering emulsion-stabilized chitosan nanocrystal films. Moreover, sharpening of the C–H stretching peak at 2920 cm^−1^ suggests increased hydrophobicity and conformational changes in the aliphatic chains due to emulsion incorporation. The shift and sharpening of the carboxylate absorption band at 1615 cm^−1^ further indicate altered local chemical environments, enhanced intermolecular interactions, and molecular rearrangement [43].

#### 3.4.3. X-Ray Diffraction (XRD) Analysis of the Films

XRD patterns (Figure 5B) showed broad features centered at 2θ ≈ 13.5° and 22.6°, consistent with a predominantly amorphous SA-rich matrix [3]. TCPE incorporation did not generate new diffraction peaks or substantial peak shifts, indicating retention of the basic SA packing pattern and the absence of a distinct new crystalline phase. The gradual increase in intensity around 22.6° may reflect greater local ordering together with a contribution from cellulose I domains in TOBC, as reported for nanocellulose-containing Pickering-emulsion films [42,43]. At moderate loading, hydrogen bonding can promote local organization, whereas electrostatic repulsion between the anionic carboxylate groups of TOBC and SA helps maintain nanofiber dispersion. The greater structural heterogeneity observed for SA-E15 by SEM indicates that excessive TCPE disrupts uniform matrix organization despite the stronger diffraction signal.

#### 3.4.4. Thermal Property Analysis of Films

TGA and DTG curves (Figure 5C,D) showed three overlapping mass-loss regions. The initial loss between 30 and 130 °C was mainly associated with free and bound water, and the greater loss of the neat SA film was consistent with its higher moisture content [44]. The major degradation region at approximately 180–275 °C included glycerol volatilization or decomposition, decarboxylation and chain scission of SA, degradation of TOBC, and loss of residual volatile CEO components. The principal DTG minimum shifted progressively toward approximately 210–220 °C after TCPE incorporation, indicating a delay in the temperature at which the maximum mass-loss rate occurred; comparable shifts have been reported for nanofiber-stabilized Pickering-emulsion films [45,46]. Above 275 °C, continued degradation and carbonization of the polysaccharide-rich matrix predominated. At the same time, the residue at 600 °C decreased as TCPE loading increased, which is consistent with a larger proportion of volatile emulsion components and a lower char yield. Therefore, TCPE altered the thermal-degradation pathway and delayed the principal DTG event, rather than uniformly improving every measure of thermal stability.

#### 3.4.5. Mechanical Properties Analysis of Films

Tensile strength (TS) and elongation at break (EAB) were used to evaluate film resistance and flexibility. SA-E10 reached the highest TS (38.56 ± 1.66 MPa, *p* < 0.05) and an EAB of 10.27%, compared with 8.09% for the neat SA film. The simultaneous improvement in strength and extensibility suggests that moderate TCPE loading balanced two effects: TOBC nanofibers reinforced the matrix through hydrogen bonding and physical entanglement, while well-dispersed emulsion droplets increased local chain mobility. Similar concentration-dependent optima have been reported for Pickering-emulsion films based on sodium alginate and other polysaccharides [38,46]. When the TCPE content reached 15%, both TS and EAB declined. This decrease agrees with the lower rheological network strength and the larger pores and structural heterogeneity observed by SEM, indicating that droplet aggregation and interfacial defects became dominant at excessive loading [47,48].

#### 3.4.6. Moisture Content (MC), Water Solubility (WS), and Water Vapor Permeability (WVP) of Films

**Figure 6:**
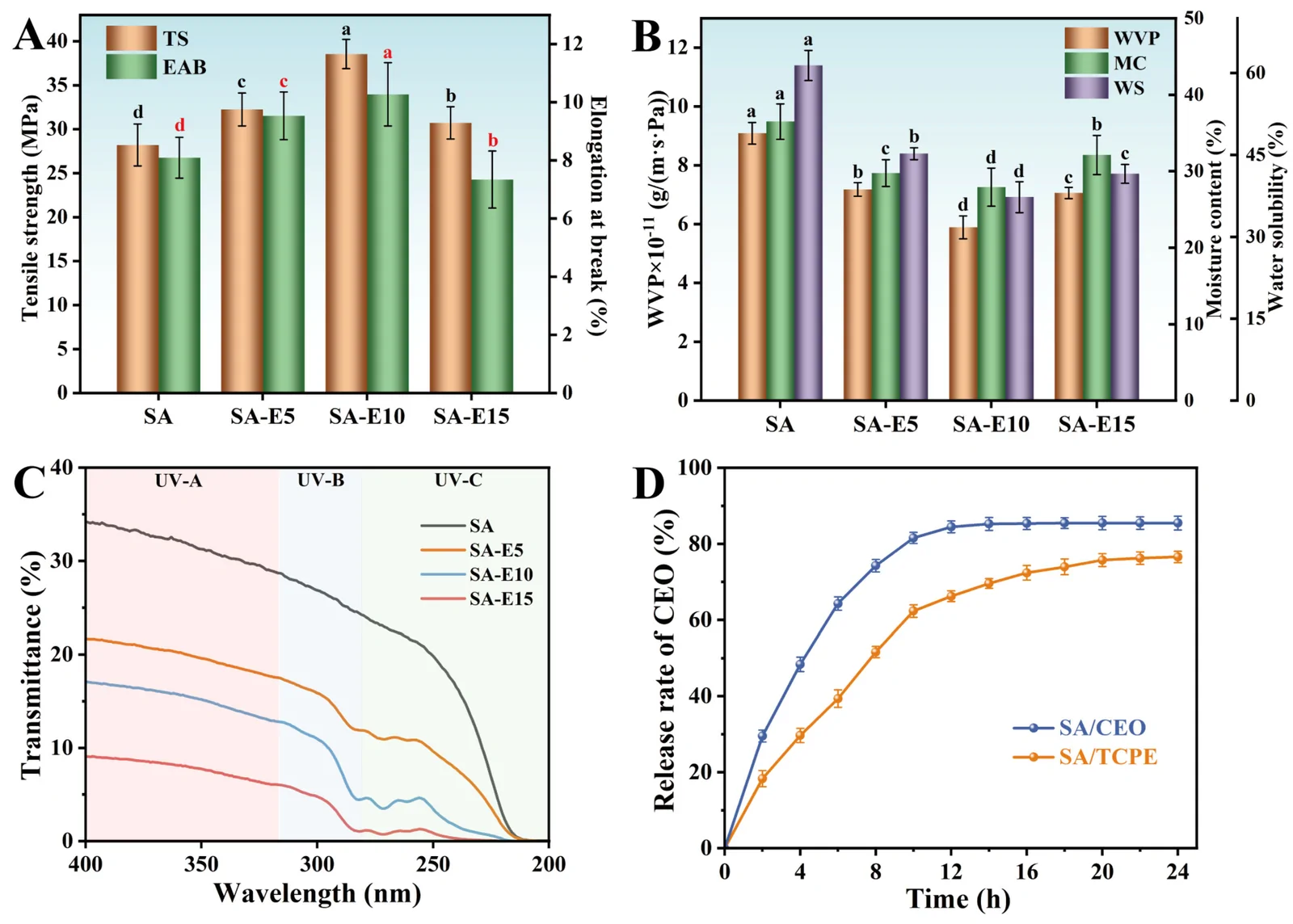
Functional properties of SA films with different TCPE loadings. (**A**) Mechanical properties, including tensile strength (TS) and elongation at break (EAB), showing the influence of TCPE concentration. (**B**) Water vapor permeability (WVP), moisture content (MC), and water solubility (WS) of films with different TCPE levels. (**C**) UV transmittance spectra across the UV-A, UV-B, and UV-C regions. (**D**) Release profiles of clove essential oil (CEO) from SA/CEO and SA/TCPE films. Different lowercase letters above the bars in panels (**A**,**B**) indicate significant differences among formulations for the same response variable (*p* < 0.05).

Water affinity and water vapor barrier behavior changed nonlinearly with TCPE loading (Figure 6B). Compared with the neat SA film, SA-E5 and SA-E10 exhibited lower WVP, with the minimum observed for SA-E10. At moderate loading, hydrophobic CEO droplets interrupt continuous hydrophilic transport pathways, while the TOBC-coated interfaces increase the tortuosity of water vapor diffusion; similar barrier improvements have been reported for other Pickering-emulsion biopolymer films [49,50]. Moisture content simultaneously decreased from 36.48 ± 2.32% for SA to 27.93 ± 0.46% for SA-E10, and water solubility also declined up to 10% TCPE, indicating lower water accessibility and stronger cohesion within the composite matrix. In contrast, WVP, MC, and WS increased when the TCPE content reached 15%. This reversal is consistent with the pores and structural heterogeneity observed by SEM: excessive droplet loading can promote coalescence and disrupt matrix continuity, creating preferential pathways for water sorption and diffusion, as reported in related systems [51,52,53,54]. Thus, barrier improvement was governed not by oil hydrophobicity alone, but by the balance between hydrophobic domains, diffusion-path tortuosity, and matrix continuity, which explains the superior overall performance of SA-E10.

#### 3.4.7. UV Resistance of Films

UV transmittance decreased across the measured 200–400 nm range as TCPE loading increased (Figure 6C). This improvement can be attributed to the aromatic structure of eugenol, which absorbs UV radiation, together with light scattering by the dispersed oil droplets and TOBC-containing interfaces. Similar concentration-dependent UV shielding has been observed in essential-oil Pickering-emulsion films [50,51]. Because the present measurement was limited to the UV region, these data demonstrate enhanced UV protection but do not, by themselves, establish that visible-light transparency was unchanged.

#### 3.4.8. Release of CEO from Films

In 50% ethanol, the film containing directly added CEO released approximately 80% of its loading within 12 h, whereas the SA/TCPE film showed a slower release and approached a plateau after approximately 20 h (Figure 6D). The TOBC layer surrounding the oil droplets and the surrounding SA matrix provide successive diffusion barriers, reducing the initial burst and increasing the path length for CEO partitioning into the release medium. A comparable retardation of essential-oil release has been reported for Pickering-emulsion-incorporated SA films [38,51]. The 50% ethanol system is a food simulant, however, and the result should be interpreted as a relative comparison of release behavior rather than direct evidence of a specific antimicrobial shelf life in real foods.

#### 3.4.9. Antioxidant and Antimicrobial Activities of Films

**Figure 7:**
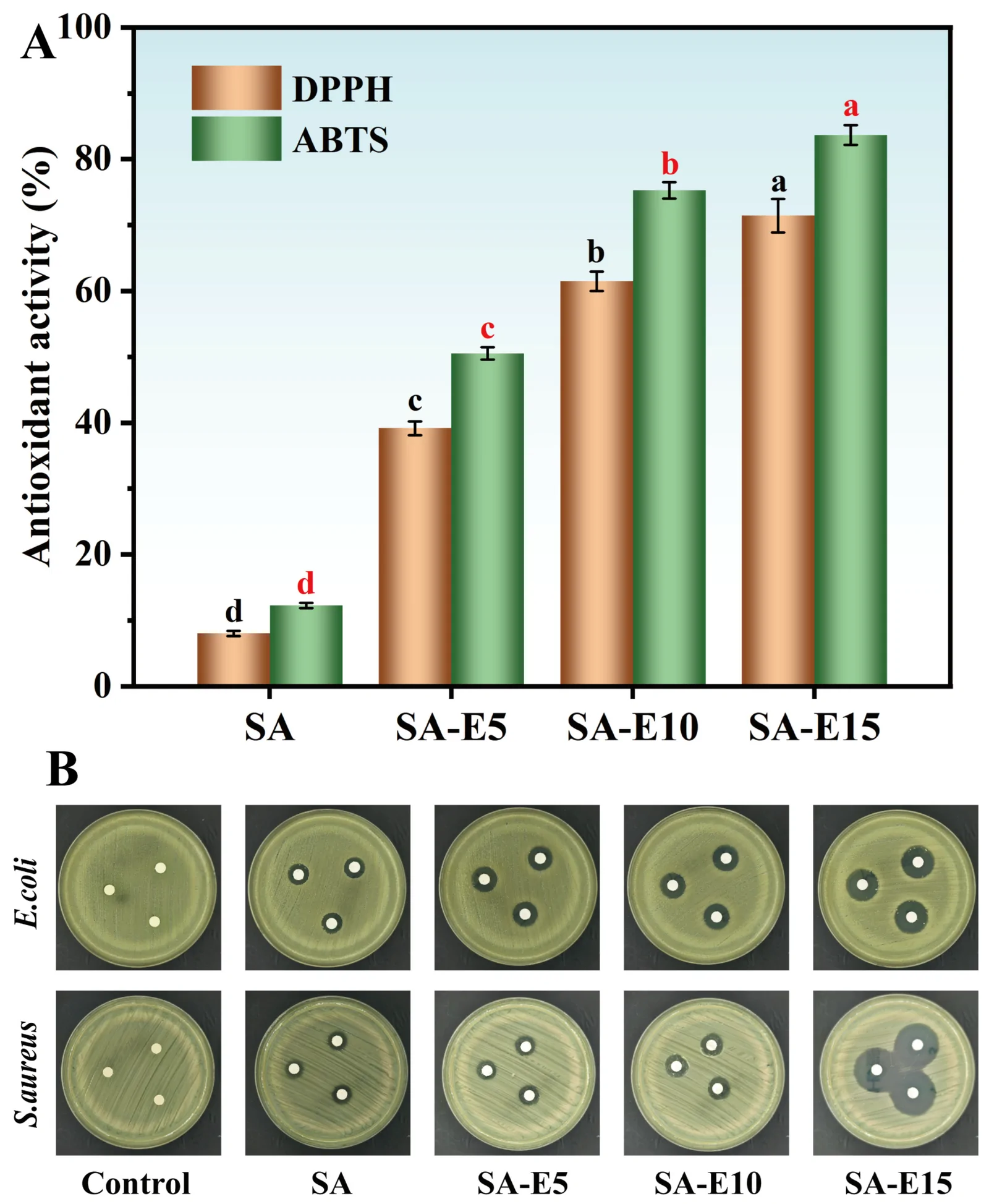
Antioxidant activity and antibacterial evaluation of SA-based systems containing different TCPE concentrations. (**A**) Antioxidant activity of the films assessed by DPPH and ABTS assays. (**B**) Agar diffusion assay of dried film-coated paper disks against *E. coli* and *S. aureus*, showing inhibition zones for each formulation. Different lowercase letters above the bars indicate significant differences among formulations within the same antioxidant assay (*p* < 0.05).

DPPH and ABTS radical-scavenging activities increased with TCPE loading, reaching 71.43 ± 2.55% and 83.66 ± 1.49%, respectively, for the highest-emulsion formulation (Figure 7A). The dose-dependent response is consistent with the increasing amount of eugenol and other phenolic constituents capable of donating hydrogen atoms or electrons to reactive radicals [48,55]. The higher ABTS than DPPH response may reflect differences in radical chemistry and in the accessibility of CEO-derived antioxidants under the two assay conditions.

Antibacterial activity against *S. aureus* and *E. coli* was evaluated using dried film-coated paper disks in the agar diffusion assay (Figure 7B). The disks coated with pure SA showed negligible inhibition, whereas TCPE-containing coatings produced visible inhibition zones that appeared to increase with emulsion loading. This response is consistent with diffusion of CEO-derived phenolic compounds, particularly eugenol, from the dried coating into the agar [6,56]. Because inhibition-zone formation depends on both antimicrobial activity and compound diffusion, the assay supports qualitative antibacterial potential but does not provide a direct measure of bacterial-killing efficiency. Together with the antioxidant results, these findings support the use of TCPE as an active component while also indicating the need for quantitative challenge tests in a packaged food system.

## 4. Conclusions

This study developed a biodegradable sodium alginate (SA)-based active packaging film containing a TEMPO-oxidized bacterial cellulose (TOBC)-stabilized clove essential oil Pickering emulsion (TCPE). TOBC provided stable interfacial coverage of CEO droplets, and moderate TCPE incorporation improved the rheological behavior of the film-forming solutions, mechanical performance, water vapor barrier properties, and UV shielding, while delaying the principal DTG mass-loss event. The films also exhibited antioxidant activity and qualitative antibacterial potential against *Escherichia coli* and *Staphylococcus aureus*. The Pickering emulsion further slowed CEO release compared with direct oil incorporation. Among the tested formulations, SA-E10 provided the best overall balance because it combined the highest tensile strength (38.56 ± 1.66 MPa), improved elongation at break (10.27%), the lowest moisture content (27.93 ± 0.46%) and WVP, as well as showing enhanced UV shielding, strong antioxidant activity, and observable antibacterial potential, while avoiding the greater heterogeneity and mechanical deterioration observed at 15% TCPE. The novelty of this work does not lie in the use of SA itself, but in coupling the interfacial stabilizing role of TOBC with its post-casting contribution to matrix organization and demonstrating how this coupled function controls the loading-dependent balance among reinforcement, barrier performance, bioactivity, and release. For industrial translation, the water-based casting system and the moderate optimal TCPE loading are favorable features; nevertheless, continuous coating or casting, controlled drying, batch reproducibility, storage stability, migration and sensory safety, real-food challenge testing, and end-of-life behavior should be evaluated before commercial food packaging application.

## Data Availability

The original contributions presented in this study are included in the article/Supplementary Material. Further inquiries can be directed to the corresponding authors.

## Supplementary Materials

The following supporting information can be downloaded at: https://www.mdpi.com/article/10.3390/foods15162924/s1, Figure S1: SEM image of bacterial cellulose (BC).
