# Supplementary material for: TEMPO-Oxidized Bacterial Cellulose-Stabilized Clove Essential Oil Pickering Emulsions for Sustained-Release Sodium Alginate Active Packaging Films
Source: Foods. 2026 Aug 20;15(16):2924. doi: 10.3390/foods15162924 (PMC13512187; doi:10.3390/foods15162924)
Supplement: Supplementary file 1 [file foods-15-02924-s001.zip › foods-4491817-supplementary.pdf]

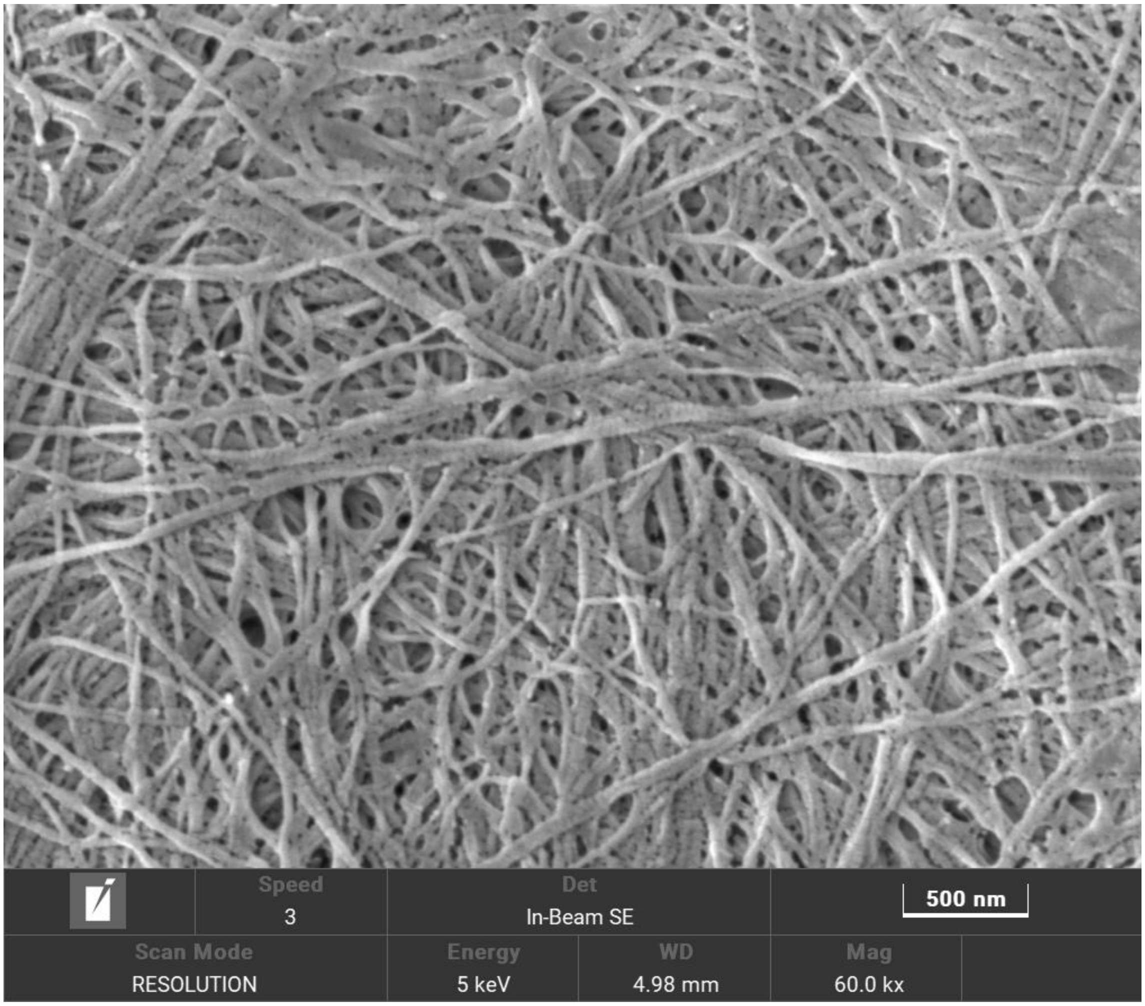

Figure S1. SEM image of bacterial cellulose (BC)
